# Supplementary material for: On the effectiveness of tracking and testing in SEIR models for improving health vs. economy trade-offs
Source: Sci Rep. 2021 Aug 11;11:16305. doi: 10.1038/s41598-021-95415-7 (PMC8357840; doi:10.1038/s41598-021-95415-7)
Supplement: Supplementary file 1 — Supplementary Information. [file 41598_2021_95415_MOESM1_ESM.pdf]

# On the Effectiveness of Tracking and Testing in SEIR Models for Improving Health vs. Economy Trade-Offs: Supplementary Information

**Yoav Kolumbus**[1,*] **and Noam Nisan**[1,+]

[1]The Hebrew University of Jerusalem, The Rachel and Selim Benin School of Computer Science and Engineering, Jerusalem, 9190401, Israel
[*]Corresponding author. Email: yoav.kolumbus@mail.huji.ac.il
[+]Email: noam@cs.huji.ac.il

# Appendix A: Geographic Small-World Networks

In the main text we present results with dynamic random graphs that are similar to the classical SEIR model, assuming uniform mixing among the population. Here we present results of experiments with a network model that takes into account geographic distances as well as small-world network effects[1]. The model assumes that vertices are located on a two-dimensional grid and interact with all their geographically close neighbors. In addition, there is a probability for each vertex to have a long-distance interaction, where the probability is inversely proportional to the square of the distance. For more details of the geographic small world network model see[1]. Here we show that the results with these networks are very similar to the results presented in the main text, indicating that even when the population mixing is not uniform but is strongly restricted by geographic distances, the basic effects of different intervention approaches and the relations between these approaches remain the same.

## Fixed Lockdowns

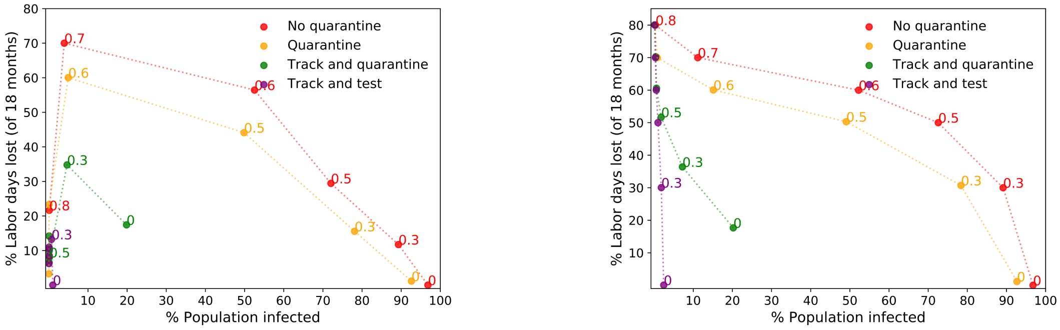


**(a)** Closed system **(b)** Open system

**Figure 1.** Tracking compared with the Quarantine method and the no-quarantine benchmark with different levels of fixed lockdowns in geographic small world networks.

## On-Off Lockdown Policies

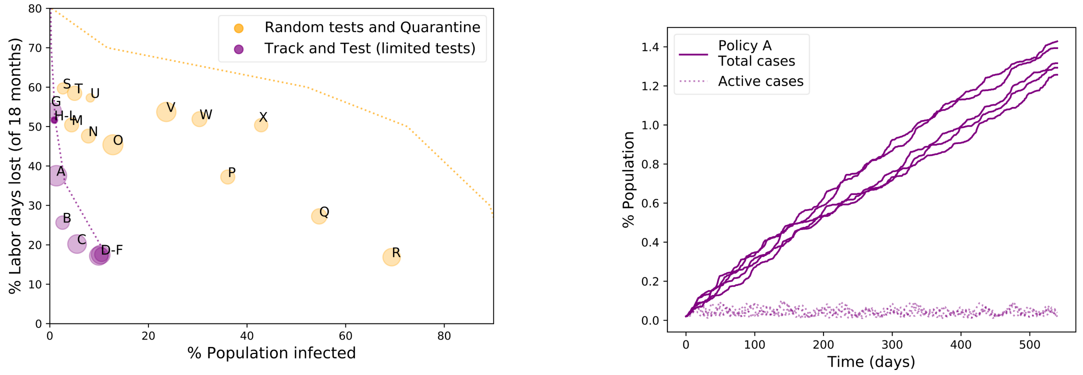


**(a)** **(b)**

**Figure 2.** On-Off policies in geographic small world networks. 2a: The cost map of the total percentage of labor days lost in 18 months versus the total percentage of population infected. The letters near the markers indicate the different policies as shown in Table 1 in the main text. Purple markers are policies with the bounded Track and Test method and orange markers are with the Quarantine method with random tests. The purple and orange dotted curves show a comparison to the results of fixed lockdown policies with bounded Track and Test and with Quarantine and random tests, respectively. 2b: Dynamics of the outbreak under policy *A* – an example of five simulation instances.

## Appendix B: Quarantine with Large-Scale Testing

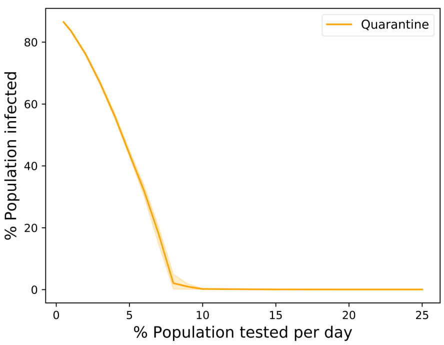


**(a)** Total infected

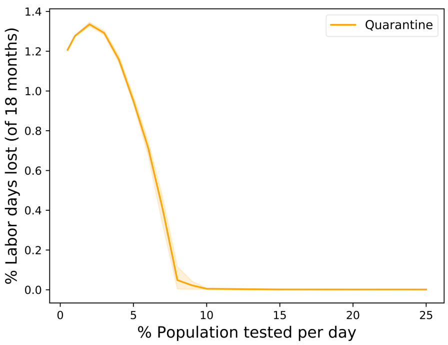


**(b)** Economic costs

**Figure 3.** Effect of large-scale testing: The Quarantine method combined with random tests with high testing capacities. Daily testing capacities are presented in percentages of the population. 3a: Total percentage of population infected as a function of the testing capacity. 3b: Economic costs as a function of the testing capacity.

## Appendix C: Track and Test with False-Negative Errors

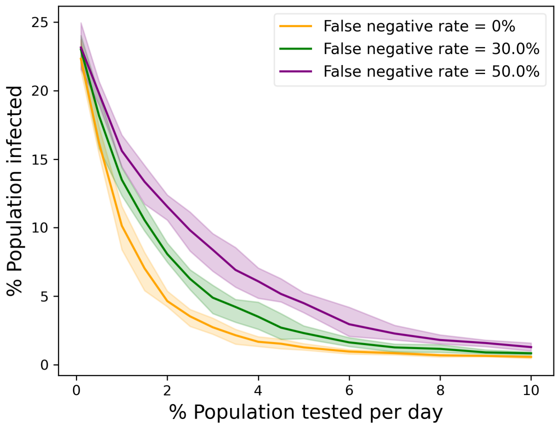


**Figure 4.** Track and test with false-negative errors: Total population percentage infected in 540 days as a function of the population-percentage capacity of daily tests under the bounded track and test method. The three colors indicate the results in three regimes of false-negative error rate, the shaded areas show the 1'st to 3'rd quartiles across simulations. The curves show that under track and test, at low testing capacities an increase in the testing capacity is highly influential even if these tests have low detection rates. Additionally, inaccuracies in tests can be compensated by an increase in the test capacity, which is inline with the model of[2]. For example, with perfect tests, track and test without any lockdown reaches less than 5% infection with a testing capacity of around 2%. In the case of a 30% false negative rate (which is more realistic to COVID) similar results are obtained with a 3% testing capacity.

## References


**1.** Kleinberg, J. The small-world phenomenon: An algorithmic perspective. In *Proceedings of the thirty-second annual ACM symposium on Theory of computing*, 163–170 (2000).

**2.** Larremore, D. B. *et al.* Test sensitivity is secondary to frequency and turnaround time for covid-19 surveillance. *MedRxiv* (2020).
